# Inactivation of *Salmonella* spp. and *Listeria* spp. by Palmitic, Stearic, and Oleic Acid Sophorolipids and Thiamine Dilauryl Sulfate

**DOI:** 10.3389/fmicb.2016.02076

**Published:** 2016-12-22

**Authors:** Xuejie Zhang, Richard Ashby, Daniel K. Y. Solaiman, Joseph Uknalis, Xuetong Fan

**Affiliations:** ^1^Institute of Vegetables and Flowers, Chinese Academy of Agricultural SciencesBeijing, China; ^2^Eastern Regional Research Center, United States Department of Agriculture – Agricultural Research ServiceWyndmoor, PA, USA

**Keywords:** sophorolipids, *Listeria monocytogenes*, *Salmonella*, thiamine dilauryl sulfate, cell membrane

## Abstract

Food contaminated with human pathogens, such as *Salmonella* spp. and *Listeria monocytogenes*, frequently causes outbreaks of foodborne illness. Consumer concern over the use of synthesized antimicrobials to enhance microbial food safety has led to a search of natural alternatives. The objectives of this study were to evaluate the antimicrobial activity of various types of sophorolipids (SLs) and thiamine dilauryl sulfate (TDS) against pathogenic *Salmonella* spp. and *Listeria* spp. Both free and lactonic forms of SLs were synthesized from *Candida bombicola* using palmitic, stearic, and oleic acids as co-feedstocks. TDS and purified SLs were used to treat cocktails of *Salmonella* spp. and *Listeria* spp. Results showed that lactonic SLs had higher antimicrobial activity than the free-acid form, and Gram-positive *Listeria* spp. were more susceptible to SLs and TDS than Gram-negative *Salmonella* spp. *Listeria* populations were reduced from an initial concentration of 7.2 log CFU/mL to a non-detectible level within a 1 min treatment of 0.1% (w/v) lactonic SLs and TDS in the presence of 20% ethanol, which itself did not significantly reduce the populations. There were no significant differences in the antimicrobial efficacy among palmitic, stearic, and oleic acid-based SLs against *Salmonella* or *Listeria* spp. Ethanol was utilized to improve the antimicrobial activity of free-acid SLs against Gram-negative bacteria. In general, TDS was more effective than the SLs against *Salmonella* and *Listeria* spp. scanning electron microscopy and transmission electron microscopy images showed that SLs and TDS damaged *Listeria* cell membranes and resulted in cell lysis. Overall, our results demonstrated that SLs and TDS in the presence of ethanol can be used to inactivate foodborne pathogens, especially Gram-positive bacteria.

## Introduction

Microbial food safety continues to be a concern in the U.S. and around the world (Center for Science in Public Interest [CSPI], 2015; Felício et al., 2015). It is estimated that foodborne pathogens lead to 48 million cases of illness, 301,000 hospitalizations and 4,300 deaths in the U.S. each year (Scallan et al., 2011a,b). The foodborne bacterial pathogens: *Salmonella* spp., *Campylobacter*, *Listeria monocytogenes*, and *Escherichia coli* O157:H7 are the most significant organisms based on the number of cases caused by these agents and/or the severity of ensuing disease. A report released by the USDA’s Economic Research Service estimates that the cost of food borne illnesses in the U.S. tops $15.6 billion annually (U.S. Department of Agriculture, Economic Research Service [USDA-ERS], 2014). *Salmonella* spp., with treatment costs estimated at $3.6 billion, is the most expensive pathogen related to cases of foodborne illness. Others topping the list include *L. monocytogenes* ($2.8 billion) and *E. coli* O157:H7 ($271 million; U.S. Department of Agriculture, Economic Research Service [USDA-ERS], 2014). Currently, synthetic additives and antimicrobials are often used by the food industry to preserve food quality and reduce microbial growth. However, consumer concerns over chemically synthesized additives have stimulated research into natural antimicrobials to improve the microbial safety of foods.

Sophorolipids (SLs), produced by a number of yeasts such as *Candida bombicola*, are a class of naturally derived glycolipid compounds containing a hydrophilic moiety and a hydrophobic moiety (Borsanyiova et al., 2016). They are biodegradable, exhibit low-toxicity and are environmentally friendly. The hydrophobic fatty acid tail and the hydrophilic carbohydrate head, sophorose, which is a glucose di-saccharide, are linked with an unusual β-1,2 bond. The carboxylic end of this fatty acid is either free (acidic or open form) or in lactonic form (internally esterified). The hydroxy fatty acids are generally 16 or 18 carbon atoms in length and may possess multiple olefinic groups. SLs have been shown to possess various antimicrobial properties against bacteria and fungi (Lang and Wagner, 1993; Kitamoto et al., 2002; Ashby et al., 2011; De Rienzo et al., 2015; Zhang et al., 2016). The antibacterial activity of SLs against *Rhodococcus erythropolis*, *Bacillus subtilis*, *Staphylococcus epidermidis*, *Streptococcus agalactiae*, *Moraxella* sp., *Pseudomonas putida*, *Enterobacter aerogenes*, and *E. coli* was evaluated by Shah et al. (2007). Results showed that SL had greater antibacterial activity against Gram-positive bacteria than Gram-negative bacteria. Sleiman et al. (2009) evaluated different SL derivatives against *E. coli* and *Staphylococcus aureus*. No significant inhibitory activity was observed for most derivatives against *E. coli* at a concentration of 512 μg/mL and against *S. aureus* at a concentration of 218 μg/mL. The antimicrobial activities of SLs against common foodborne pathogens, however, have not been evaluated except in our earlier publication on inactivation of *E. coli* O157:H7 (Zhang et al., 2016).

It is known that physicochemical and biological properties of SLs are significantly influenced by the distribution of the lactone vs. free-acid forms in the fermentative broth (Van Bogaert et al., 2007; Sleiman et al., 2009; De Rienzo et al., 2015; Maddikeri et al., 2015). The specific fatty acids used in the growth medium also influence the composition of SLs. It is unclear whether changing fatty acid composition in SLs will have any influence on their antimicrobial activities.

Thiamine dilauryl sulfate (TDS), a vitamin B1 derivative, has been approved for use as a food additive in Japan. Fransisca et al. (2012) reported that a combination of 1% TDS with 10% malic acid was effective in reducing the *E. coli* O157:H7 population on alfalfa seeds. Our earlier results (Zhang et al., 2016) showed that TDS was the most effective among several types of tested bio-surfactants.

Previous studies have shown that SLs cause morphological changes on the cell surface of bacteria (De Rienzo et al., 2015). However, the mechanisms involved in SL- and TDS-mediated inactivation of pathogenic Gram-positive bacteria remain unclear even though it has been proposed that SLs act at the cell membrane level. Our earlier results (Zhang et al., 2016) showed that the outer membrane of *E. coli* cells treated with SL derived from oleic acid and TDS separated from the plasma membrane. The effects of SLs on membranes have not been tested against Gram-positive bacteria which have distinctive cell wall structures.

The objectives of the study were to evaluate the antimicrobial activities of various types of SLs and TDS against *Salmonella* spp. and *Listeria* spp., and elucidate the mechanism for the antimicrobial effects of SL’s against *Listeria* spp.

## Materials and Methods

### Bacteria Strains and Propagation

Pathogenic *S. Enteritidis* ATCC 13076, *S. Stanley* H0558, and *L. monocytogenes* Scott A 724, and non-pathogenic *L. innocua* 33090, *L. innocua* 33091, and *L. innocua* 51742 were obtained from USDA, ARS, ERRC culture collections. The non-pathogenic strains of *Listeria* are often used as surrogates for *L. monocytogenes* in studies because they behave similarly and have similar characteristics as *L. monocytogenes*.

Stock cultures were maintained at -80°C. Cultures of each individual strain were propagated on tryptic soy agar (TSA, Difco Laboratories, Detroit, MI, USA.) at 37°C and maintained at 4°C until use. Prior to the inoculum preparation, individual *Salmonella* and *Listeria* cells were grown in 10 mL tryptic soy broth (TSB: Difco Laboratories) for 18–20 h. After centrifugation at 1,800 ×*g* for 10 min, the cultures were suspended in 10 mL phosphate buffered saline (pH 7.2). The two *Salmonella* strains, and four *Listeria* cultures were combined to form two cocktails of cultures.

### Biosynthesis and Purification of Palmitic, Stearic, and Oleic Acid SLs

Sophorolipids were synthesized from *C. bombicola* ATCC 22214 using palmitic, stearic, and oleic acids as co-feedstocks, followed by extraction and purification (Ashby et al., 2011; Solaiman et al., 2015). Briefly, 10 L of *Candida* growth medium (CGM; 10% w/v glucose, 1% w/v yeast extract, and 0.1% w/v urea) was prepared in a 12-L capacity vessel of a bench-top bioreactor (Bioflo 3000 Batch/Continuous Bioreactor, New Brunswick, NJ, USA). Following sterilization by autoclaving and then cooling to 26°C, palmitic, stearic, and oleic acids (200 mL, technical grade, Sigma–Aldrich, St. Louis, MO, USA) were added to the medium to a final concentration of 2% (v/v). A stock inoculum culture (previously prepared and stored in -80°C freezer) was thawed and added to the 10-L CGM (containing 2% palmitic, stearic, or oleic acids) medium to initiate the fermentation. The bioreactor was set at the following parameters: 26°C, an impeller speed of 700 rpm, 2 L/min aeration, and no pH control. On day 2 of the fermentation, 7.5% (w/v) of granular glucose and 2% (v/v) of palmitic, stearic, or oleic acids were added to the culture. On day 5, 1% (v/v) of palmitic, stearic, or oleic acids was again added. On day 7, the entire culture (cells and broth) was collected and lyophilized in ∼2-L volumes (for ease of handling). The dried residues were transferred to Erlenmeyer flasks and extracted in excess ethyl acetate with shaking at room temperature for 2 days. The extract was filtered through Whatman No. 2 filter paper. The solids on the filter were additionally rinsed with two volumes of ethyl acetate to recover any residual SLs. The ethyl acetate fractions containing the SLs were combined and concentrated using a rotary-evaporator. The concentrate was slowly added to excess hexane to obtain the pure SLs in crystallized form. Each of the parental SL mixtures was analyzed by LC/MS according to the procedure previously outlined by Nuñez et al. (2004) and in all cases were found to be composed of greater than 95% lactones.

### Free-Acid SL Synthesis

**FIGURE 1:**
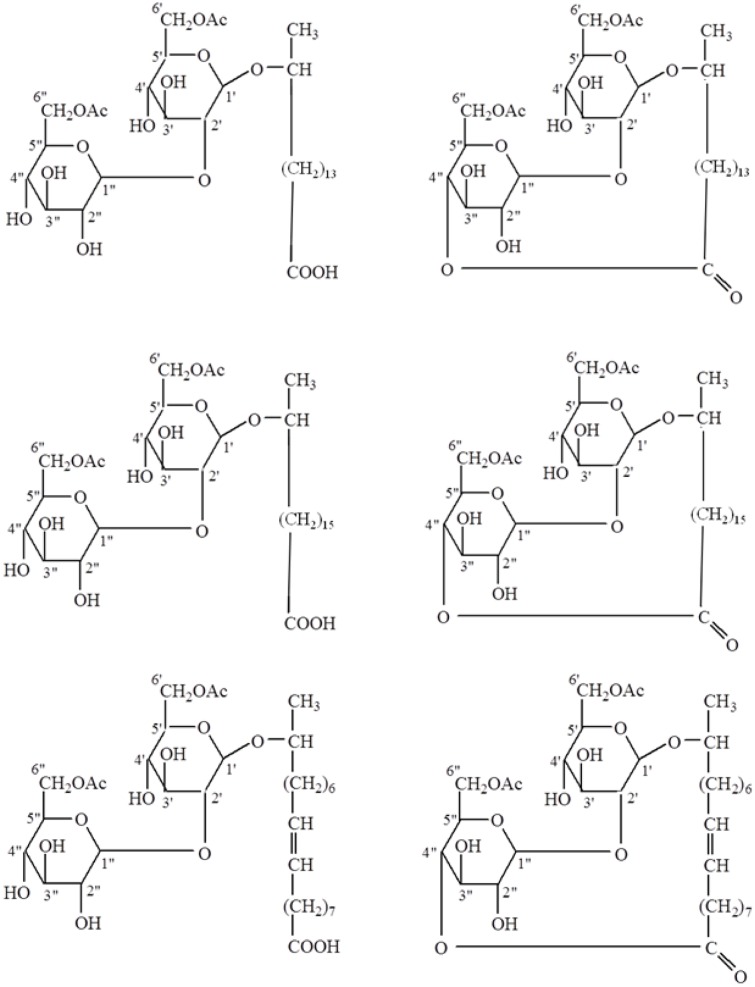
**Structures of free-acid** **(A,C,E)** and lactonic **(B,D,F)** types of palmitic **(A,B)**, stearic **(C,D)**, and oleic **(E,F)** acid-based sophorolipids.

Free-acid SLs were produced by base-catalyzed ring-opening chemical reactions of the lactone form. A six molar solution of potassium hydroxide (KOH) was prepared in deionized water and 10 g of each lactonic SL (three total) was added to the KOH solution. This mixture was stirred overnight at room temperature, heated to 40°C and stirred for an additional 3 h. The mixture was then acidified to pH 3–4 using phosphoric acid and poured into a crystallization dish where a steady stream of nitrogen was used to help remove the water. Desalting was accomplished by stirring the dried SL mixture with excess 2:1 CHCl_3_:ethanol and placing the mixture in a stoppered flask to inhibit CHCl_3_ evaporation. After a few hours celite was added to the mixture (helps in filtration, otherwise the fine salts clog the filtration frit) and the free-acid SLs were recovered by vacuum filtration through Whatman #2 filter paper. The solids were washed an additional two times with 2:1 CHCl_3_: ethanol, and the free-acid SLs were recovered and dried under vacuum until constant weight. In total, six SLs were obtained with three in the free-acid form and three in the lactonic form (**Figure 1**).

### Antimicrobial Test

Purified palmitic, stearic, and oleic acid SLs in both free-acid and lactonic forms and TDS (Kanematsu U.S.A. Inc., Somerset, NJ, USA) at concentrations of 0.1, 0.5, and 1.0% (w/v) were tested in suspensions of *Salmonella* and *Listeria* cocktails. Stock solutions (5%, w/v) of palmitic, stearic, and oleic acid SLs and TDS were first dissolved in 100% ethanol. The stock solutions were then diluted in sterile water or ethanol to final concentrations of 0.1, 0.5, and 1.0% and various levels of ethanol (1–20%). Due to low solubility, 0.1, 0.5, and 1.0% of SLs and TDS could only be dissolved in minimum levels of 1, 5, and 10% ethanol, respectively.

For the antimicrobial test, 10 μL aliquots of bacterial inoculum were added into 990 μL of surfactant solutions giving a cell density of ∼7.2 log CFU/mL. The mixtures were then vortexed for 10 s. After 1 min and 1 h at 22°C, the suspensions were immediately serially diluted with neutralizing buffer (Difco Laboratories), and aliquots (100 μL or 1 mL) were spread plated on TSA. Plates were incubated at 37°C for 24 h and the number of colonies of bacteria was recorded. In a separate experiment, the effect of other treatment times (1 min, 1, 2, and 4 h) was also studied. Detection limit was 1 log CFU/mL. Propagation of bacteria and antimicrobial tests were carried out in a BSL-2 laboratory.

### Scanning Electron Microscopy (SEM) and Transmission Electron Microscopy (TEM) of *Listeria* spp.

For the preparation of SEM samples, 100 μL aliquots of *Listeria* spp. inoculum were mixed with 900 μL of water, 20% ethanol, 1% TDS, or 1% stearic acid SL. The concentrations of TDS and stearic acid SL were chosen based on results in previous tests and the solubility of the compounds. Aliquots (20 μL) of the mixtures were placed onto acetone-cleaned 12 mm round cover glass slips (Thermo Scientific, Portsmouth, NH, USA) and allowed to adhere for 30 min. Samples were then covered with 50 μL of 2.5% glutaraldehyde [Electron Microscopy Sciences (EMS), Hatfield PA, USA] and were allowed to fix for 30 min. Samples were then rinsed twice for 30 min each with 2–3 mL 0.1 M imidazole buffer (EMS), and dehydrated in 2 mL ethanol solutions (50, 80, 90, and 100% sequentially). After dried using ethanol, the samples were placed in a Critical Point Drying Apparatus (Denton Vacuum, Inc., Cherry Hill, NJ, USA) using liquid carbon dioxide (Welco Co, Allentown, PA, USA) for approximately 20 min. The samples were mounted onto stubs and sputter gold coated for 1 min (EMS 150R ES), followed by viewing with a FEI Quanta 200 F Scanning Electron Microscope, (Hillsboro, OR, USA) at an accelerating voltage of 10 KV in high vacuum mode.

For TEM examination, *Listeria* cells were suspended in a 2.5% glutaraldehyde solution (EMS) after treatment with water, 20% ethanol, 1% TDS, or 1% stearic acid SL. The samples were then centrifuged to pellet the cells, and the cells were re-suspended in 10 μL of warm 1% agarose (FMC Bioproducts, Rockland, ME, USA). The pellets were then washed with 0.1 M imidazole solution for 30 min, and fixed with 100 μL 1% osmium tetroxide solution (EMS) in a fume hood for 1 h. The cells were re-suspended and the samples were allowed to stand for 1 h, and dehydrated using graded ethanol solutions of 50, 80, 90, and 100%. Ethanol was then replaced with propylene oxide twice for 5 min. EMbed-812 (EMS) was mixed and used at a 50–100 % solution with propylene oxide starting with 500 μL 50 % mixture. The EMbed 812 plastic was cured in a vacuum oven (VWR, Wayne, PA, USA) at 60°C and 25 inch Hg overnight. Thin sections at approximately 70 nm were cut using a Reichert Ultracut S (Leica, Wien, Austria) with a Diatome Ultra 45° diamond knife (Fort Washington, PA, USA). Sections were collected on a copper 200 mesh grid (EMS) and stained with 1% solution of uranyl acetate (EMS) for approximately 1 min, followed by rinses with water and counter stain with lead citrate for 1 min. Thin sections were observed using a Philips Transmission Electron Microscope CM 12 (Philips, Netherlands) with an accelerating voltage of 80 KV, and imaged with a DVC detector and processed with AMT software (Danvers, MA, USA).

### Statistical Analysis

Treatments were repeated three times for each experiment. Data were statistically analyzed using general linear model (GLM) of SAS 8.1 (SAS Institute Inc., Cary, NC, USA). Differences between the means were separated using the Bonferroni–Dunn test (*P* < 0.05).

## Results And Discussion

### Comparison of Free and Lactonic SLs against *Salmonella* spp.

**FIGURE 2:**
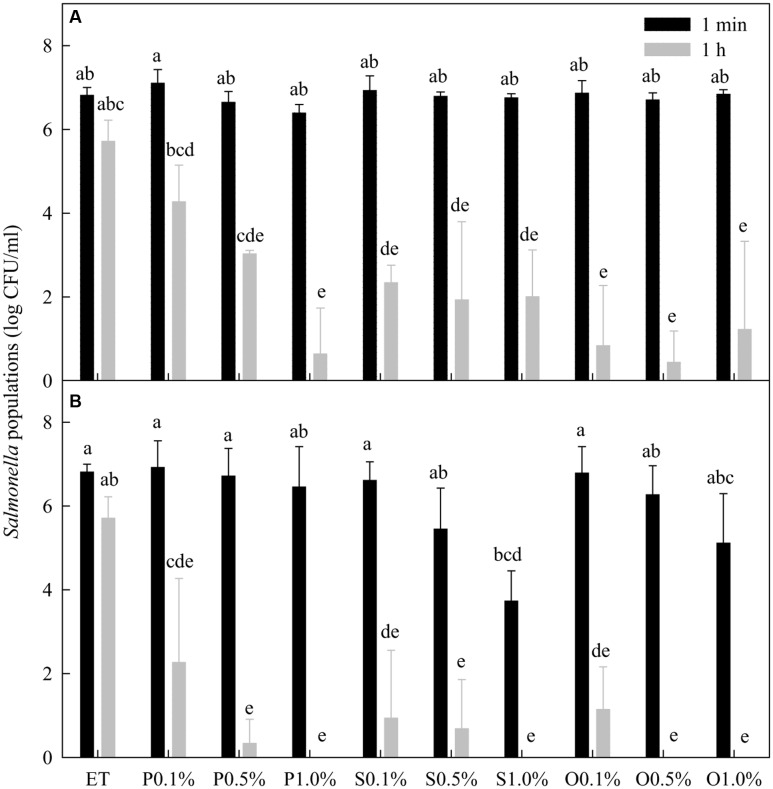
**Effect of free-acid** **(A)** and lactonic **(B)** palmitic (P), stearic (S), and oleic (O) acid sophorolipids on *Salmonella* populations. Palmitic, stearic, and oleic acid sophorolipids at 0.1, 0.5, and 1.0% were prepared in 20% ethanol (ET). The sophorolipid solutions were mixed with a cocktail of *Salmonella* spp. After 1 min and 1 h treatment, survival *Salmonella* populations were determined. Means with the same letter are not significantly different (*P* > 0.05). Vertical bars represent standard deviations (*n* = 3).

Ethanol at 20% did not significantly (*P* > 0.05) reduce the population of *Salmonella* spp. within 1 h of contact at 22°C (**Figure 2**). Within 1 min treatment, none of the free-acid SLs in the presence of 20% ethanol significantly reduced the population of *Salmonella* spp. in any of the three concentrations (0.1, 0.5, and 1.0%) tested. After 1 h, however, all free-acid SLs at all three concentrations significantly lowered *Salmonella* populations. There was no significant (*P* > 0.05) difference among the three concentrations of free-acid stearic and oleic SLs in their effectiveness in reducing the populations of *Salmonella* while free-acid palmitic acid SL reduced *Salmonella* populations in a concentration dependent manner.

In general, lactonic SLs were more effective than free-acid SLs in reducing the *Salmonella* populations (**Figure 2**). For the 1 min treatment, 1% lactonic stearic SL reduced populations of *Salmonella* by 3.1 logs while other treatments at any concentration did not significantly reduce the populations. After 1 h treatment, all lactonic SLs at the three concentrations tested significantly (*P* < 0.05) reduced *Salmonella* populations. Compared with 20% ethanol, more than 5 log reductions of *Salmonella* were achieved by all three lactonic SLs at all concentrations after 1 h except that palmitic acid SL at 0.1% reduced the *Salmonella* population only by 4.1 log CFU/mL.

Thiamine dilauryl sulfate at 0.1% in the presence of 20% ethanol reduced *Salmonella* populations from 7.3 log CFU/mL to non-detectable level after 1 min of treatments (data not shown).

### Effect of Ethanol on the Efficacy of SLs against *Salmonella* spp.

**FIGURE 3:**
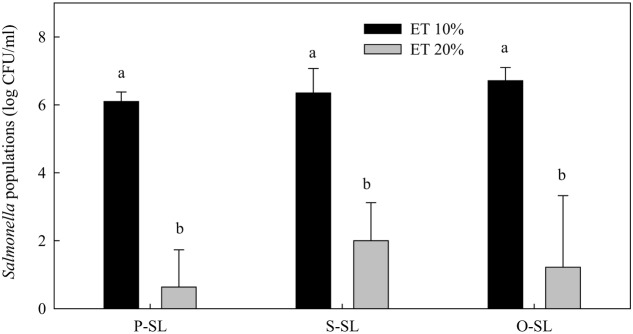
**Effect of ethanol concentration on the effectiveness of free-acid palmitic (P-SL), stearic (S-SL), and oleic (O-SL) acid sophorolipids in inactivating *Salmonella*.** Sophorolipids at 1% level were prepared in 10 or 20% ethanol (ET). After *Salmonella* cells were treated with the sophorolipids for 1 h, survival *Salmonella* populations were determined. Means with the same letter are not significantly different (*P* > 0.05). Vertical bars represent standard deviations (*n* = 3).

In the presence of 10% ethanol, none of the free-acid SLs at 1% significantly reduced *Salmonella* population after 1 h incubation (**Figure 3**). In the presence of 20% ethanol, significant reductions of *Salmonella* were achieved regardless of the fatty acid type. The results indicate that relatively high concentrations of ethanol were needed for SLs to significantly reduce *Salmonella* populations.

### Effects on *Listeria* spp.

**FIGURE 4:**
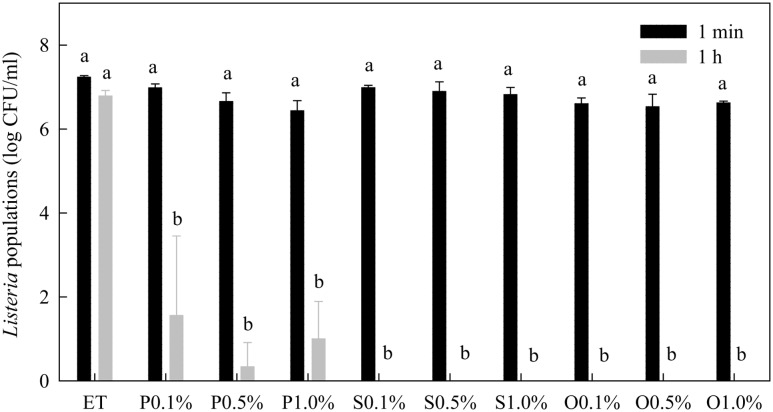
**Effect of free-acid palmitic (P), stearic (S), and oleic (O) acid sophorolipids on *Listeria* population.** Sophorolipids at 0.1, 0.5, and 1.0% were prepared in 20% ethanol. The solutions were mixed with a cocktail of *Listeria* spp. *Listeria* populations were determined after 1 min and 1 h of treatment. Means with the same letter are not significantly different (*P* > 0.05). Vertical bars represent standard deviations (*n* = 3).

Within 1 min treatment, none of the free-acid SLs significantly affected the populations of *Listeria* spp. (**Figure 4**). However, after 1 h treatment time, the populations of *Listeria* were significantly reduced by all three types of free-acid SLs at all three concentrations tested. The populations of *Listeria* were reduced to the non-detectable level (1 log CFU/mL) by most of the treatments. Compared with their effects on *Salmonella* (**Figure 2**), the free-acid SLs were more effective against *Listeria* after 1 h of treatment. Ethanol itself at 20% did not significantly reduce the populations of *Listeria* spp. within 1 h.

**FIGURE 5:**
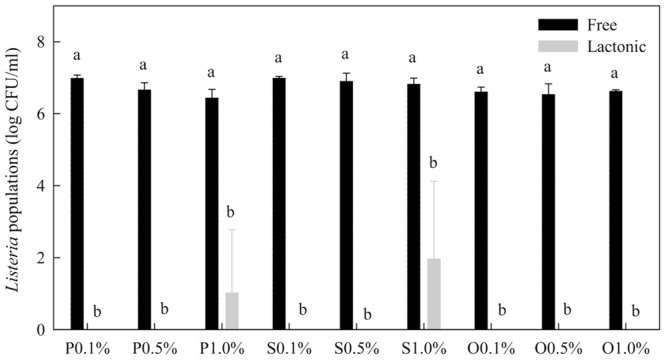
**Comparison of free-acid and lactonic palmitic (P), stearic (S), and oleic (O) acid sophorolipids in inactivating *Listeria* spp.** Sophorolipids at 0.1, 0.5, and 1.0% in the presence of 20% ethanol were mixed with *Listeria* cells. After 1 min, survival *Listeria* populations were determined. Means with the same letter are not significantly different (*P* > 0.05). Vertical bars represent standard deviations (*n* = 3).

Lactonic SLs were much more effective than free-acid SLs (**Figure 5**). After 1 min treatment, free-acid SLs in the presence of 20% ethanol only reduced *Listeria* population by 0.2–0.8 log CFU/mL while lactonic SLs reduced the populations by more than 5 logs. Initial *Listeria* population before treatments was 7.2 log CFU/mL.

**FIGURE 6:**
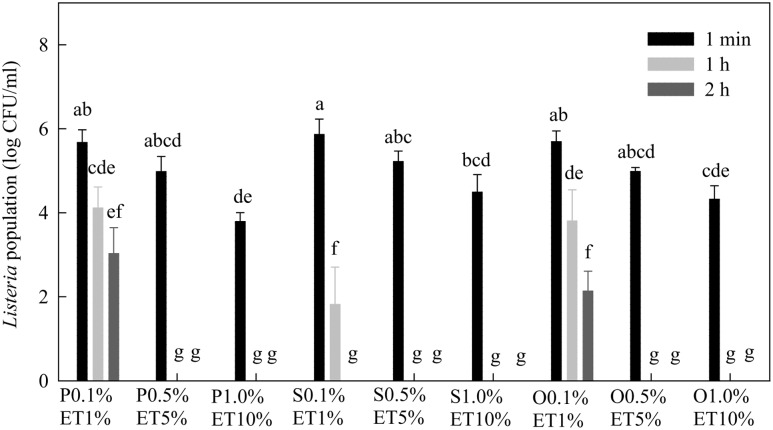
**Effect of lactonic palmitic (S), stearic (S), and oleic (O) acid sophorolipids on *Listeria* populations.** Sophorolipids with concentrations of 0.1, 0.5, and 1.0% were prepared in 1, 5, and 10% ethanol (ET), respectively, and used to treat a cocktail of *Listeria* spp. for 1 min, 1 h, and 2 h. Means with the same letter are not significantly different (*P* > 0.05). Vertical bars represent standard deviations (*n* = 3).

After 1 min treatment, all three lactonic SLs at 0.1% in the presence of 1% ethanol reduced *Listeria* population by about 0.4–0.5 log CFU/mL (**Figure 6**). Lactonic SLs at 0.5% in 5% ethanol and 1.0% in 10% ethanol reduced populations of *Listeria* by 2.0–2.2 and 2.8–3.4 log CFU/mL, respectively. Increasing contact time (treatment time) from 1 min to 2 h reduced *Listeria* populations. At 0.5 and 1.0%, lactonic SLs reduced *Listeria* populations to non-detectable levels after 1 or 2 h of treatment. Stearic acid SL was more effective against *Listeria* than palmitic or oleic acid SLs as evidenced at 0.1% after 1 and 2 h treatments.

Thiamine dilauryl sulfate at 0.1% in the presence of 20% ethanol reduced *Listeria* population to a non-detectable level after 1 min of treatment (data not shown). In the presence of 1% ethanol, 0.1% TDS reduced *Listeria* populations by 3.6 log CFU/mL. Our results suggested that TDS was more effective than the SLs against *Salmonella* and *Listeria* spp., similar to our earlier results on *E. coli* O157:H7 (Zhang et al., 2016).

### Cell Morphology of *Listeria* spp.

**FIGURE 7:**
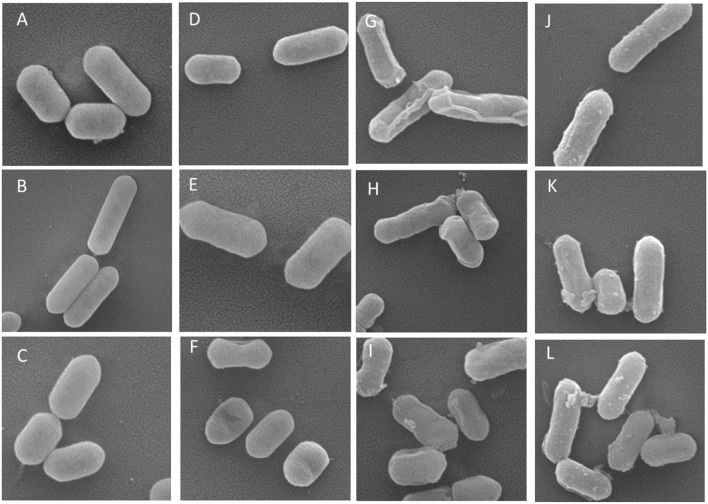
**Representative scanning electron microscopy (SEM) images of *Listeria* cells treated with water** **(A–C)**, 20% ethanol **(D–F)**, 1% of lactonic oleic acid sophorolipid **(G–I)**, and 1% TDS **(J–L)** for 2 h. Magnifications were 50,000×.

The SEM images showed that non-treated *Listeria* cells had typical plump rod shapes with smooth surfaces and various lengths (**Figure 7**). A few cells treated with 20% ethanol exhibited some shrinkages and depressions. Most lactonic oleic acid SL-treated cells were highly distorted, and had corrugated surfaces with folds, shrinkages, lumps, and protuberances. TDS-treated cells had lumps and protuberances on the surface. Unlike cells treated with SL, TDS-treated cells did not show any folds or shrinkages. The distorted and corrugated surfaces of cells treated with SLs may be results of the cell lysis and leakage of cellular contents while the lumps and protuberances on the surface of TDS-treated cells may be indicative of cellular leakages (Dengle-Pulate et al., 2014).

**FIGURE 8:**
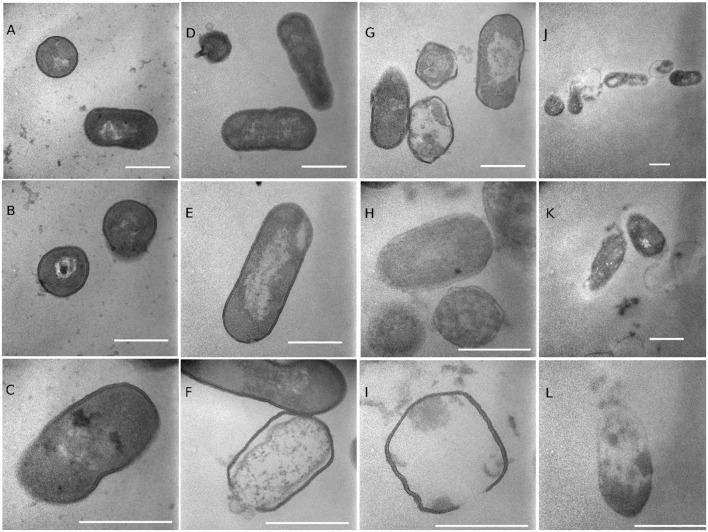
**Transmission electron microscopy (TEM) images of *Listeria* cells treated with water** **(A–C)**, 20% ethanol **(D–F)**, 1% of lactonic oleic acid sophorolipid **(G–I)**, and 1% TDS **(J–L)** for 2 h. The markers represent 500 nm.

Transmission electron microscopy images showed the morphology of non-treated and treated *Listeria* cells (**Figure 8**). The cell walls and membranes of non-treated *Listeria* cells were mostly intact, showing well-preserved, dense cell contents. Cells treated with 20% ethanol had a similar morphology as the non-treated cells with occasional lysed cells. Oleic acid SL-treated cells had less defined cell membranes, and often with broken walls and membranes, and increased heterogeneity in electron density in the cytoplasm. It appears that some TDS-treated cells underwent lysis, resulting in the release of their cellular contents into the surrounding environment. It was common to find electron-dense particles or precipitates around damaged bacterial cells that were electron translucent in comparison to undamaged cells (Jung et al., 2008). Our earlier study (Zhang et al., 2016) on *E. coli* O157:H7 showed that SL and TDS-treated cells had lumps and protuberances on their surface. Similar SL-induced morphological changes (lumps and protuberances) of bacteria cells were observed by other researchers (Dengle-Pulate et al., 2014). TEM images of *E. coli* O157:H7 also revealed that the outer membrane of *E. coli* cells treated with SLs and TDS were separated from the plasma membrane (Zhang et al., 2016). Dengle-Pulate et al. (2014) observed, using SEM, that SLs synthesized using lauryl alcohol acted on the integrity of *E. coli* and *S. aureus* cell membranes, and the treated cells were disrupted with the outpouring of their cytoplasmic contents, which leads to cell lysis.

Our results demonstrated that Gram-positive *Listeria* spp. were more sensitive to SLs and TDS than Gram-negative *Salmonella* spp. The results are in agreement with several earlier reports on other bacteria (Kim et al., 2002; Shah et al., 2007; Dengle-Pulate et al., 2014). Dengle-Pulate et al. (2014) found that SLs synthesized using lauryl alcohol showed complete inhibition against *E. coli* at 30 μg/mL at a contact time of 2 h whereas for *S. aureus*, complete inhibition was observed at 10 μg/mL. Kim et al. (2002) reported that SLs showed antimicrobial activity against Gram-positive bacteria: *B. subtilis*, *S. xylosus*, *S. mutans*, and *Propionibacterium acne*, but no effect on *E. coli*. Reetika et al. (2012), however, found that linolenic SL mixture (containing 80% lactonic form) was more potent against Gram-negative bacteria than Gram-positive ones, with minimum inhibitory concentrations (MICs) of 20 and 10 μg/mL for *B. subtilis* and *E. coli*, respectively. Pulate et al. (2013) showed that SLs, produced using glucose as a hydrophilic source and lauryl alcohol C12–14 as a hydrophobic source by *C. bombicola*, had potent antimicrobial activity against both *E. coli* and *S. aureus*. The difference in antimicrobial activity of TDS and SLs against Gram-positive and Gram-negative microorganisms may be attributed to the structure of their respective cell walls. The cell wall of Gram-negative microorganisms is comprised of a thin peptidoglycan layer and two layers of membranes which control the eﬄux of substance across the membranes. In contrast, the cell wall of Gram-positive microorganisms is composed of one layer of membrane (cytoplasmic membrane). The cytoplasmic membrane is surrounded by a thick peptidoglycan layer. The peptidoglycan of the cell is sufficiently porous to allow the passing of compounds with molecular mass less than 50 kDa (Lang and Wagner, 1993). The molecular mass of SLs used in our study are typically 500–800 Da and maintain amphiphilic properties, which can allow SLs to enter the peptidoglycan layer and interact with the cytoplasmic membrane (Dengle-Pulate et al., 2014). Our results showed that TDS and SLs caused morphological changes and damage to the membranes of *Listeria* spp. which might be the cause of cell death.

Palmitic, stearic, and oleic acids were used as fermentation co-substances to synthesize three fatty acid SLs with different lengths (C18 vs. C16) and saturation (saturated and unsaturated). Palmitic acid is the most common fatty acid found in animals, plants, and microorganisms while oleic acid and stearic acid also occur naturally in various animal and vegetable fats and oils. It seems that there is no difference among the three fatty acid SLs in their antimicrobial activities with exception of stearic acid SL which was more effective against *Listeria* spp. than oleic and palmitic acid SLs at 0.1% after 1 and 2 h of treatments (**Figure 6**). An earlier study (Shah et al., 2007) found that SLs obtained from different sugar-containing media differed in their activities against several microorganisms including *B. subtilis*. R. *erythropolis*, *S. epidermidis*, and *Moraxella* spp. SLs from arabinose-containing medium were more effective against three of the four Gram-positive bacteria tested and against *Moraxella* sp. than SLs from glucose-containing medium. However, SLs from arabinose showed no inhibition of the growth of *E. coli* while SLs from lactose-containing medium were the most effective compound against *B. subtilis*.

Sophorolipids and TDS are generally insoluble in water, but soluble in organic solvents such as ethanol. Therefore, to incorporate SLs and TDS into water, the compounds were first dissolved in ethanol before dilution. Our results showed that ethanol itself at concentrations up to 20% had no significant effects on populations of *Listeria* or *Salmonella* spp. within 1 h of treatment. Our results further demonstrated that 20% of ethanol was needed for the antimicrobial effects against *Salmonella* spp. of free SLs at the concentrations tested. De Rienzo et al. (2016) reported that growth of Gram-negative *Cupriavidus necator* and Gram-positive *B. subtilis* were inhibited by undefined SLs only at high concentrations (5%). The presence of caprylic acid (0.8% v/v) enhanced the inhibition of biofilm formation of *P. aeruginosa*, *E. coli*, and *B. subtilis*. Kim et al. (2002) reported that SLs had no effect on *E. coli*. Sleiman et al. (2009) also reported that SLs did not show any significant antibacterial activity *in vitro* when tested at clinically relevant concentrations. The primary target of ethanol is the cell membrane (Ingram, 1990), and ethanol is known to inactivate bacteria by disrupting cell membrane, and serving as a membrane perturbant (Fried and Novick, 1973). Ethanol may enhance the penetration of SLs and TDS through lipid bilayers of bacteria, especially Gram-negative bacteria which had two membranes (cytoplasmic and outer membranes). Our results suggested that ethanol enhanced antimicrobial activity of SLs against *Salmonella* and *Listeria*, and there was a synergistic interaction between SLs and ethanol.

Our results show that SLs, particularly in lactonic form, were effective in inactivating *Salmonella* and *Listeria* spp. in bacterial suspension. It is envisioned that the compounds may be used in different ways to minimize the risk of pathogen contamination in various foods. For example, SLs may be incorporated into ready-to-eat meat or cheese products to reduce the populations of *L. monocytogenes*, a pathogen often associated with the products. Furthermore, the compounds may be used as a wash to minimize pathogen cross-contamination for the handling and processing of fresh and fresh-cut produce. With their strong surfactant property, the antimicrobial SLs could be valuable in the formulation of washing and cleaning solutions. In addition, the compounds may be used as a coating on various foods. However, since SLs and TDS require the presence of 10–20% ethanol for effective antimicrobial activity against *Salmonella* and *Listeria*, their commercial applications on foods may be limited. One viable possibility may be for disinfecting food contact surface. Furthermore, the compounds may be combined with other antimicrobials to achieve synergistic effects against human pathogens. The actual effectiveness and applications of these compounds in food need further investigation.

In summary, we synthesized six types of SLs from *C. bombicola* by using palmitic, stearic, and oleic acids in growth media, and evaluated their antimicrobial activity against two *Salmonella* and *Listeria* spp. Our results showed that lactonic SLs were more effective antimicrobials than free SLs, and *Listeria* spp. were more sensitive to SLs and TDS than *Salmonella* spp. TDS was a more effective antimicrobial than the SLs. In addition, ethanol enhanced antimicrobial activities of TDS and SLs. Overall, our results demonstrate that SLs and TDS are effective antimicrobials and may be used to inactivate foodborne pathogens.

## Notes

Mention of trade names or commercial products in this publication is solely for the purpose of providing specific information and does not imply recommendation or endorsement by the U.S. Department of Agriculture. USDA is an equal opportunity employer and provider.

## Author Contributions

XZ conducted most of the experiments and participated in the preparation of manuscript writing. RA and DS participated in the synthesis of the compounds and the preparation of manuscript writing. JU conducted SEM studied and participated in the preparation of manuscript writing. XF conceived ideas, conducted data and statistical analysis, and prepared manuscript.

## Conflict of Interest Statement

The authors declare that the research was conducted in the absence of any commercial or financial relationships that could be construed as a potential conflict of interest.
